# Efficacy of Daily Walking as a Potential Predictor of Improved Health-Related Quality of Life in Patients with Type 2 Diabetes in Korea

**DOI:** 10.3390/healthcare12161644

**Published:** 2024-08-18

**Authors:** Wonil Park, Dongjun Lee

**Affiliations:** 1 Physical Activity and Performance Institute, Konkuk University, 120 Neungdong-ro, Gwangjin-gu, Seoul 05029, Republic of Korea; wonilpark01@konkuk.ac.kr; 2 Division of Sports, College of Arts and Physical Education, Myongji University, 116 Myongji-ro, Cheoin-gu, Yongin 17058, Republic of Korea

**Keywords:** health-related quality of life, walking, physical activity, frequency, diabetes

## Abstract

This study examines whether health-related quality of life (HRQoL) scores differ due to the frequency of walking or physical activity (PA) throughout a week in diabetic patients in Korea. This population-based cross-sectional study used data from the 2018–2021 Korea National Health and Nutrition Examination Survey. The dependent variable was HRQoL scores as measured with EuroQol-5D (EQ-5D). The independent variables were defined as three types of PA: (1) walking; (2) moderate; and (3) vigorous. An estimated population size of 2,376,066 was included in this study. The mean (95% confidence interval (CI)) age of patients was 60.12 years (59.23, 60.81), and 53.0% were female. The mean (95% CI) of EQ-5D was 0.867 (0.857, 0.877). The majority of diabetic patients reported walking daily (39.05%, 95% CI; 36.28–41.81%), while a significant proportion did not engage in moderate (65.45%, 95% CI; 62.79–68.11%) or vigorous (78.38%, 95% CI; 73.02–77.73%) PA at all. After controlling for covariates, EQ-5D scores significantly increased when patients had walked once per week for at least 10 min in the Tobit regression model. The frequency of walking was the most significant predicting factor for better HRQoL in patients with type 2 diabetes.

## 1. Introduction

Diabetes mellitus has become a widespread epidemic and health problem in the world [1]. Also, it is well known that diabetes and its complications are the fifth most common reason for death among Korean adults, related to an elevated risk of cardiovascular mortality and morbidity [2]. The total number of diabetic patients in Korea has risen between six- and seven-fold during the previous 40 years. Currently, as of 2020, it is estimated that almost 16.7% of Korean people over 30 years of age or older have diabetes or pre-diabetes [3], which is a state wherein blood glucose levels are above normal. According to the recently released Diabetes Fact Sheet in Korea by the Koren Diabetes Association, there are approximately 6 million diabetic patients over the age of 30 in Korea as of 2020. This statistic is nearly 30 years ahead of the projection made in the Diabetes Fact Sheet in 2012, which estimated that the number of diabetic patients would rise to 5.91 million by 2050 [4].

Quality of life (QoL) is a person’s evaluation of satisfaction with life in relation to their culture and morals. It is intricately influenced by an individual’s physical and mental well-being, degree of independence, and social connections [5]. A strategy that takes into account physical, mental, and social aspects is recognized as health-related QoL (HRQoL) [6]. Evaluating HRQoL is a crucial part of any healthcare assessment [7]. In particular, diabetes and its complications can induce impaired HRQoL as well as cardiovascular diseases such as heart disease and stroke. In addition, individuals with diabetes have decreased levels of disease progression and complications in their HRQoL [8]. Most studies investigating the results of complications in diabetes on QoL have shown that those who have diabetes have significantly worse HRQoL associated with complications [9,10,11]. There is no question that cost-effective strategies for managing diabetes could provide economic benefits [12] as well as relieve pain and improve the QoL of adult patients with diabetes [13]. Cost-effective diabetes management strategies should undoubtedly offer economic benefits [12], alleviate pain, and enhance the QoL of adults with diabetes [13].

Physical activity (PA) is measured by the number of days and duration that exercise is conducted for at least ten minutes, including walking at moderate and vigorous intensity. Here, walking is generally classified as low-to-moderate in intensity. Moderate-intensity PA refers to activities (e.g., carrying light loads, swimming at a regular pace, and doubles tennis) that require moderate effort and cause individuals to “breathe somewhat harder than normal”. Vigorous-intensity PA refers to activities that require substantial effort and cause individuals to “breathe much harder than normal” [14]. Most of all, PA is the cornerstone of lifestyle modification in type 2 diabetes, which is associated with cardiovascular risks and morbidities [15]. Habitual PA or exercise intervention is related to improved glycemic control and glucose intolerance, and it may delay the progression of diabetes [16]. Regular PA has a significant impact on the QoL of patients with diabetes, particularly in the areas of physical health and disease management [17]. Furthermore, several studies have demonstrated that regular PA positively impacts both the physical and mental aspects of HRQoL in patients with diabetes and the elderly [18,19,20]. Potential mechanisms underlying the relationship between PA and QoL or well-being include PA-induced changes in brain neurotransmitters and endogenous opioids, which are linked to depression, anxiety, and other mood-related factors [21,22]. Additionally, research has shown that reductions in psychological distress encompassing depression, anxiety, stress, and sleep disturbances are associated with improvements in well-being and life satisfaction [23]. Also, typical forms of PA, including yoga, tai chi, and aquatic training, can improve HRQoL and mental health in adult patients with type 2 diabetes [16]. Cugusi and colleagues have reported on the association between aquatic-based exercise and HRQoL in men with type 2 diabetes. This study demonstrated that HRQoL showed significant improvement in post-assessments compared to baseline measurements [24].

Although participation in PA is a strategy that can prevent or postpone the deterioration effects of diabetes, most patients with diabetes are not active. However, it is unknown whether the frequency of PA is associated with the adverse impacts of diabetes on HRQoL. Therefore, our study sought to assess the correlation between the frequencies of PA and HRQoL in adult patients with diabetes in Korea with data taken from the 2018–2021 Korea National Health and Nutrition Examination Survey (KNHANES).

## 2. Materials and Methods

### 2.1. Participants and Procedures

The current study employed data from the 2019 to 2021 Korea National Health and Nutrition Examination Survey (KNHANES). The Korea Centers for Disease Control and Prevention Agency (KCDA) conducts nationally representative KNHANES annually. KNHANES is a continuous surveillance system that has started to generate national data about the health conditions, health-related behaviors, and food and nutrient intake of the Korean population [25]. The KNHANES data were provided through the Center for Disease Control and Prevention, Division of Health and Nutrition Survey (https://knhanes.kdca.go.kr/knhanes/sub03/sub03_02_05.do) (accessed on 3 January 2024). The current study focused on non-institutionalized Korean residents who were over 19 years old and had reported diabetes. The sample was clustered via multiple stages, using a clustered design of probability with primary sampling units (PSUs), households within PSUs, and individuals aged 1 year and over within households. The KNHANES consists of three major components: (a) an interview on health, (b) a medical assessment, and (c) a nutrition analysis. Among those components, the first component (health interview) was utilized for the current study.

### 2.2. Measurements

The dependent variable was HRQoL scores, as measured with EuroQol-5D (EQ-5D). The Korean version of EQ-5D was applied to diabetic patients to evaluate its validity and reliability in a previous study [26]. The EQ-5D variables were defined in two different ways [27]. First, the total EQ-5D score was included in the analyses as the continuous variable with a range of 0–1. Second, all five items (mobility, self-care, usual activity, pain/discomfort, and anxiety/depression) were included in the analyses as dichotomous variables (without problems and with problems). The independent variables included three types of physical activity: (1) walking (light); (2) moderate; and (3) vigorous activity [28]. The physical activity survey was conducted using the face-to-face interview method [25]. First, the walking variable was defined as the number of days that a participant walked for a minimum of 10 min per week. Second, the moderate physical activity variable referred to the number of days that the respondent did at least 10 min of more strenuous or breathless moderate physical activity than usual in a week. Third, the vigorous physical activity variable was specified as the number of days in a week that the attendant engaged in at least 10 min of much more strenuous or breathless physical activity than usual. In the present study, demographic information such as age (19–64 or 65+), gender (male or female), region (city or non-city), household income (low, lower middle, upper middle, or high), education (elementary school or lower, middle school, high school, and university or higher), and the presence of comorbid diseases were included as covariates.

### 2.3. Data Analysis

Overall, the consequences of the sample design were integrated into our analysis of the complex survey data for the estimator’s accuracy. Descriptive statistics were used to describe the characteristics of patients with diabetes in Korea and the EQ-5D scores based on their physical activities. Multiple logistic regression models were implemented to estimate the likelihood of the patient’s responsiveness to each of the five dimensions of EQ-5D using the frequency of physical activity while controlling for described covariates. Multiple Ordinary Least-Squares (OLS) regression and Tobit regression models were conducted to weigh the impact of physical activity on EQ-5D scores after controlling for covariates. The models of Tobit regression were utilized because a significant number of remarks was at the upper limit, and the dependent variable’s distribution was censored and skewed. An α level for statistical significance of 0.05 was used. The Statistical Analysis System (SAS) version 9.4 was used for all analyses (SAS Institute Inc., Cary, NC, USA).

## 3. Results

### 3.1. Population Characteristics

The characteristics of adult patients with diabetes in Korea are shown in Table 1. A total of 2405 patients were identified and given a diabetes diagnosis across the study years. The average annual weighted number of patients was 2,376,066. The mean (95% confidence interval (CI)) age of patients was 60.12 years (59.23, 60.81), and 52.97% were female. The majority of patients with diabetes reported that they walked every day (39.05%, 95% CI, 36.28–41.81%), while they did not engage in moderate or vigorous physical activities at all (65.45% (95% CI, 62.79–68.11%) and 75.38% (95% CI, 73.02–77.73%), respectively). Approximately 60% of patients reported that they had a cardiovascular disease.

### 3.2. EQ-5D Scores Based on Physical Activity

Table 2 shows the EQ-5D scores based on physical activity among diabetic patients in Korea. Regarding three variables (walking (mild), moderate, and vigorous physical activities), the results indicate that the EQ-5D score of patients who did not perform physical activity for at least ten minutes per day were the lowest. Also, the EQ-5D score of patients who walked a minimum of 10 min or who undertook moderate and vigorous PA in a week was the second lowest. The mean (95% CI) of the EQ-5D was 0.867 (0.857, 0.877). In terms of walking, the EQ-5D score of patients who never walked for at least 10 min at a time in a week was 0.78, which is the lowest. Also, the EQ-5D scores of adult patients with diabetes who walked for at least ten minutes per day was 0.87, which is the second-lowest score. However, the range of EQ-5D scores in adult patients with diabetes who walked for at least ten minutes per day from 1 to 6 days a week ranged from 0.87 to 0.93. 

Table 3 shows that these aforementioned findings pertain to the response of each of the five features of EQ-5D in the multiple logistic regression analysis. The patient’s age, gender, education level, household income, and the presence of several comorbid diseases were included in the model to assess the likelihood of having problems related to each of the five aspects of EQ-5D according to the frequency of physical activities. 

First, regarding the mobility dimension, compared to patients who never walked for at least 10 min in a week, the likelihood of having problems related to mobility for the patients who walked over 10 min for at least one day in a week was lower (adjusted OR: 0.40 to 0.86). A similar pattern was found concerning moderate physical activity. Compared to patients who did not undertake moderate physical activity for at least ten minutes per day, the likelihood of having problems related to mobility for patients who performed over ten minutes of moderate or vigorous PA, at least one time a week, was lower (adjusted OR: 0.41 to 0.83). However, a related pattern between vigorous physical activity and the likelihood of having problems related to mobility was not found (adjusted OR: 0.56 to 1.55). 

Second, generally, there was a moderate relationship between the three types of PAs and the likelihood of having problems related to self-care.

Third, the usual activity dimension also indicated a pattern similar to the mobility dimension. Overall, walking and moderate activity had a connection to the likelihood of having problems related to usual activities (adjusted OR: 0.57 to 0.65 and adjusted OR: 0.50 to 0.95), but vigorous activity did not have an association with the likelihood of having problems related to usual activities (adjusted OR: 0.69 to 1.42, respectively), except for groups with patients who walked or undertook moderate physical activity for at least 10 min a day for 5 days in a week.

Fourth, walking is linked to a higher chance of pain/discomfort in relation to usual activity (adjusted OR: 0.44 to 0.80), but a connection was not found between moderate and vigorous activities and the likelihood of having problems (pain/discomfort) related to usual activity (adjusted OR: 0.41 to 1.67 and adjusted OR: 0.84 to 2.14). 

Fifth, the anxiety/depression dimension also showed a pattern similar to the mobility and usual care dimension.

In general, walking and moderate activity were connected to the likelihood of having problems related to usual activity (adjusted OR: 0.70 to 0.92, (except for groups that had patients who walked or undertook moderate physical activity for at least 10 min a day for 5 to 7 days in a week) and adjusted OR: 0.60 to 0.95 (except for groups in which patients undertook moderate physical activity for 5 days in a week), respectively), but vigorous activity did not have an association with the likelihood of having problems related to usual activity (adjusted OR: 0.74 to 1.93).

Table 4 shows the influence of PA on EQ-5D scores in adult patients with diabetes in Korea after controlling for covariates. Overall, EQ-5D scores were significantly increased by as many as 0.04–0.11 points when patients with diabetes walked once per week for at least ten minutes per day, compared to those who never walked, based on Tobit regression. However, performing vigorous physical activity was not remarkably associated with better EQ-5D points in the Tobit regression model (Tobit coefficients range: −0.06 to 0.02). In addition to physical activities, age (seniors), lower income, and lower education levels were statistically significant predictors related to lower EQ-5D scores.

## 4. Discussion

We present a study using data from the KNHANES to explore the association between walking or PA frequency and HRQoL in diabetic patients over 19 years of age in Korea. Our study underscores the importance of promoting walking or moderate PA as a cost-effective approach to enhancing HRQoL and managing type 2 diabetes effectively.

Since walking was the most common method for adult patients with diabetes in our study to perform PA, walking duration was given to calculate PA levels. One of the most popular PAs for middle-aged and older adults is walking, which is simple to include in daily life [29]. There are numerous studies demonstrating daily walking’s meaningful effects on reducing the risk of type 2 diabetes, cardiovascular disease, and mortality [30]. Several studies have determined the substantial advantages of accustomed engagement with moderate-intensity PA, like walking, for both overall health and the management of a diversity of diseases [14,31]. Our study confirms that changes in the HRQoL aspects of physical capability, mental stability, endurance, and social interactions in older adults are favorably correlated with increased PA [32]. In other cross-sectional studies, HRQoL in diabetes is shown to decrease with lower levels of PA [20]. Given these findings, the results of this study can be generalized and applied using data from the KNHANES, which includes a large number of subjects and implements strict quality management for a large community-based group. Additionally, EQ-5D scores, which are based on walking, which is the most common form of daily PA, were highest among those who walked more frequently (walking 1–6 days/week: EQ-5D range = 0.87–0.93). This is consistent with previous research, indicating that daily walking is positively associated with HRQoL in older adults and patients with chronic kidney disease [33,34]. Similarly, research conducted in Urban Health Centres Europe (UHCE) demonstrated a longitudinal association between PA and HRQoL among community-dwelling older adults. This study showed that even moderate increases in PA frequency over time were linked to significant improvements in both physical and mental HRQoL [35]. This is because PA helps in managing blood glucose levels, reducing the risk of complications, and improving mood and energy levels. Moreover, engaging in regular PA is associated with reduced symptoms of depression and anxiety, which are common in diabetic patients [36]. Therefore, encouraging daily walking in patients with diabetes can help maintain better health outcomes.

In multiple logistic regression models, walking and moderate activity on mobility, usual activity, and anxiety/depression dimensions were notably linked to reduced problems in adult patients with diabetes. However, vigorous activity did not have any significant relation with mobility (adjusted OR: 0.56 to 1.55). There was an association between the three types of PA and the likelihood of having problems related to self-care in general. Walking was associated with lower problems in the pain/suffering dimension. However, moderate and vigorous activities were not found in any relationship. Therefore, our multiple logistic regression analysis found that the EQ-5D index improved with walking over 10 min/week. Diabetic patients were more likely to have issues in each of the five areas where physical activity was lower. Interestingly, vigorous PA was not incorporated with all of the dimensions in this study except the self-care dimension. These results show a discrepancy compared to the research findings of Abonie and colleagues, in which vigorous-intensity activities were inversely correlated to anxiety and worry (r = −0.20; *p* < 0.05, respectively) [37]. On the other hand, compared to vigorous forms of PA, waking, which is categorized as affordable and safe, may be simpler, more authentic, and obtainable for diabetic patients [38]. Walking has been linked in this study to an increased QoL, which implies that walking-based physical activity may be advantageous for adult patients with diabetes. However, recent studies show that adults with diabetes do not meet the recommended exercise amount, and it is difficult to motivate individuals to change their lifestyle habits [20]. Therefore, physical activity plays a key role in HR-QoL beyond the context of precaution and the treatment of long-term diseases. In addition, in-depth research is needed to examine physical activity and HRQoL for diabetes and patients who are hospitalized or rehabilitated.

A study that evaluated the factors affecting the EQ-5D index in adult patients with diabetes in Korea reported on gender, age, insurance type, education level, occupational status, economic status, smoking, drinking, exercise, and chronic diseases [39]. In this study, older age, region (city), lower house income, and lower education level were linked to lower EQ-5D scores. This corresponds to a Chinese study in which age, education level, chronic conditions, short-term housing, unemployment, deprivation, and absence of habitual physical activity were all related to poorer EQ-5D index outcomes [40].

Exercise interventions, whether they be aerobic, resistance training, or both, help diabetic patients not only improve their insulin sensitivity and glucose regulation but also reduce the complications associated with diabetes and enhance quality of life [41]. Although there were no notable differences in the HRQoL of those at risk for diabetes regarding glucose tolerance [42], exercise training has a positive effect on HRQoL rather than a direct effect on improving diabetes [43]. In this study, walking or physical activity of at least 10 min/week showed a significant association with HRQoL, and consistent research results were also shown in intervention studies [44,45]. Therefore, the consequences of this study demonstrate the noteworthiness of satisfying QoL through PA within the diabetic population and can be the basis for clinical practice guidelines.

This study has several limitations. First, since this was a cross-sectional study design, a direct cause-and-effect relationship between PA and HRQoL could not be established in adult patients with diabetes due to the self-reported measurements. Second, it is challenging to recall precisely the duration and intensity of PA. Third, due to the use of data from the National Health and Nutrition Examination Survey, it was not possible to select the diabetic patients, which is more related to our assessment of PA and self-management, and all PAs closely related to HRQoL were not considered. In addition, this study revealed a relationship between PA and HRQoL in diabetic patients only, which cannot be compared with the healthy population. However, while previous studies have been limited in suggesting physical activity or lower QoL in diabetes, this study can reveal the importance of daily physical activity for controlling social, demographic, and disease-related factors.

## 5. Conclusions

In summary, this study demonstrates a compelling issue and the positive impact of regular walking on HRQoL among diabetic patients in Korea. This finding emphasizes the need for public health initiatives to promote and support regular physical activity, especially walking, as part of comprehensive diabetes management strategies, aiming to mitigate the cost of diabetes and its correlated complications on patients with diabetes and their quality of life. Therefore, this study can help healthcare professionals create more targeted interventions, informing patient education, treatment planning, and public health policies to ensure physical activity is a key component in diabetes management.

## Figures and Tables

**Table 1:** Characteristics in patients with diabetes in Korea (unweighted N = 2405; weighted N = 2,376,066).

Variable	Unweighted N	Weighted N	Percentage (95% CI)
Age (yrs)			
Adult (19–64)	1189	1,465,639	61.68 (59.31, 64.06)
Elderly (>65)	1216	910,428	38.31 (35.94, 40.69)
Sex			
Male	1150	1,258,594	52.97 (50.36, 55.58)
Female	1255	1,117,472	47.03 (44.42, 49.63)
Region			
City	1406	1,289,305	54.26 (51.20, 57.32)
Non-city	999	1,086,761	45.74 (42.68, 48.80)
House-income			
Low	886	755,197	32.63 (30.05, 35.22)
Lower middle	609	604,291	26.11 (23.57, 28.66)
Upper middle	448	490,965	21.22 (18.88, 23.56)
High	409	463,752	20.04 (17.70, 22.38)
Education			
Elementary school or lower	1246	1,096,971	46.39 (43.71, 49.08)
Middle school	364	368,888	15.60 (13.62, 17.58)
High school	516	594,814	25.16 (22.67, 27.65)
College or higher	270	303,859	12.85 (11.01, 14.69)
Cardiovascular comorbidity			
No	776	855,966	36.04 (33.42, 38.65)
Yes	1628	1,519,412	61.35 (61.35, 66.58)
Musculoskeletal comorbidity			
No	1658	1,715,967	72.24 (69.93, 74.55)
Yes	746	659,411	27.76 (25.45, 30.07)
Respiratory comorbidity			
No	2098	2,101,041	88.43 (86.98, 89.87)
Yes	307	275,025	11.57 (10.13, 13.02)
Endocrine comorbidity			
No	2298	2,276,974	95.83 (94.84, 96.82)
Yes	104	95,816	4.03 (3.05, 5.01)
Cancer comorbidity			
No	2269	2,275,255	95.86 (94.94, 96.78)
Yes	133	98,264	4.14 (3.22, 5.06)
Mental comorbidity			
No	1977	2,004,222	84.41 (82.68, 86.13)
Yes	427	370,214	15.59 (13.87, 17.32)
Skin comorbidity			
No	2338	2,315,381	97.52 (96.79, 98.24)
Yes	66	59,056	2.49 (1.76, 3.12)
Urologic comorbidity			
No	2370	2,340,370	98.56 (98.00, 99.13)
Yes	34	34,066	1.43 (0.87, 2.00)
Digestive comorbidity			
No	2323	2,287,686	96.35 (95.35, 97.34)
Yes	81	86,750	3.65 (2.66, 4.64)

Note. CI = confidence interval.

**Table 2:** EQ-5D scores based on physical activity in patients with diabetes in Korea.

Frequency of Physical Activity	Walking		Moderate Activity		Vigorous Activity	
	Mean	95% CI	Mean	95% CI	Mean	95% CI
0	0.78	(0.75, 0.81)	0.85	(0.84, 0.86)	0.85	(0.84, 0.87)
1	0.90	(0.87, 0.92)	0.91	(0.90, 0.94)	0.90	(0.86, 0.95)
2	0.91	(0.88, 0.93)	0.92	(0.90, 0.94)	0.94	(0.93, 0.96)
3	0.90	(0.88, 0.92)	0.92	(0.89, 0.95)	0.90	(0.88, 0.93)
4	0.88	(0.83, 0.93)	0.90	(0.87, 0.95)	0.94	(0.92, 0.97)
5	0.90	(0.88, 0.92)	0.89	(0.86, 0.92)	0.90	(0.86, 0.94)
6	0.93	(0.91, 0.96)	0.94	(0.91, 0.98)	0.91	(0.87, 0.95)
7	0.87	(0.85, 0.88)	0.87	(0.85, 0.90)	0.89	(0.85, 0.93)

Note. The frequency of physical activity is referred to as days. The definition of ‘moderate activity’ is an activity that requires moderated physical effort, which evokes a somewhat increased heart rate or breathing, and ‘vigorous activity’ is an activity that requires hard physical effort and evokes a large increase in heart rate or breathing. EQ-5D = Euro-Quality of Life 5 Dimension and CI = confidence interval.

**Table 3:** The impact of physical activity on the response of each of the five dimensions in EQ-5D in adult patients with diabetes in Korea (multivariate logistic regression models; control for covariates).

Frequency of Physical Activity (Days/Week)	Walking		Moderate Activity		Vigorous Activity	
	Adjusted OR	95% CI	Adjusted OR	95% CI	Adjusted OR	95% CI
Mobility						
1 vs. 0	0.86	0.46, 1.59	0.75	0.46, 1.22	1.09	0.50, 2.39
2 vs. 0	0.75	0.42, 1.34	0.69	0.31, 1.50	0.56	0.26, 1.19
3 vs. 0	0.66	0.41, 1.07	0.83	0.44, 1.55	1.29	0.53, 3.13
4 vs. 0	0.61	0.33, 1.13	0.41 ^a^	0.18, 0.94	0.45	0.13, 1.58
5 vs. 0	0.66	0.37, 1.17	0.78	0.40, 1.53	1.55	0.64, 3.74
6 vs. 0	0.40 ^b^	0.21,0.75	0.47	0.18, 1.20	0.73	0.18, 2.87
7 vs. 0	0.76	0.55,1.06	0.73	0.46, 1.56	1.09	0.54, 2.21
Self-care						
1 vs. 0	0.49	0.23, 1.00	0.89	0.43, 1.83	0.94	0.37, 2.37
2 vs. 0	0.46 ^a^	0.23, 0.89	0.37 ^b^	0.17, 0.77	0.28 ^a^	0.08, 0.99
3 vs. 0	0.38 ^b^	0.20, 0.71	0.38	0.14, 1.02	0.57	0.22, 1.51
4 vs. 0	0.51	0.23, 1.14	0.50	0.07, 3.47	0.03 ^b^	0.00, 0.25
5 vs. 0	0.96	0.43, 2.13	0.33	0.11, 1.01	0.68	0.11, 4.36
6 vs. 0	0.36 ^a^	0.15, 0.86	0.35	0.08, 1.57	0.84	0.25, 2.87
7 vs. 0	0.44 ^c^	0.30, 0.66	0.71	0.42, 1.20	1.55	0.67, 3.62
Usual activity						
1 vs. 0	0.62	0.35, 1.08	0.72	0.40, 1.28	1.23	0.63, 2.43
2 vs. 0	0.59	0.35, 1.02	0.95	0.54, 1.67	0.69	0.29, 1.68
3 vs. 0	0.65	0.39, 1.09	0.50 ^a^	0.28, 0.92	1.13	0.57, 2.24
4 vs. 0	0.59	0.30, 1.16	0.64	0.27, 1.52	0.78	0.21, 2.87
5 vs. 0	1.12	0.58, 2.15	1.17	0.53, 2.58	1.02	0.29, 3.55
6 vs. 0	0.57	0.30, 1.07	0.59	0.23, 1.50	0.76	0.27, 2.16
7 vs. 0	0.87	0.62, 1.23	0.92	0.60, 1.40	1.42	0.75, 2.67
Pain/suffering						
1 vs. 0	0.69	0.41, 1.16	0.59	0.34, 1.01	1.80	1.00, 3.25
2 vs. 0	0.67	0.40, 1.12	1.03	0.60, 1.76	0.94	0.50, 1.76
3 vs. 0	0.73	0.47, 1.12	0.82	0.48, 1.41	2.14 ^a^	1.10, 4.17
4 vs. 0	0.57	0.32, 1.00	1.27	0.65, 2.48	1.25	0.43, 3.66
5 vs. 0	0.87	0.50, 1.51	1.67	0.66, 4.25	1.01	0.46, 2.19
6 vs. 0	0.44 ^a^	0.20, 0.98	0.41 ^a^	0.18, 0.94	0.86	0.23, 3.18
7 vs. 0	0.80	0.58, 1.10	1.05	0.68, 1.63	0.84	0.45, 1.58
Anxiety/depression						
1 vs. 0	0.70	0.36, 1.33	0.88	0.49, 1.59	1.16	0.54, 2.48
2 vs. 0	0.83	0.42, 1.63	0.95	0.51, 1.78	1.93 ^a^	1.03, 3.61
3 vs. 0	0.79	0.48, 1.30	0.76	0.40, 1.47	1.11	0.55, 2.26
4 vs. 0	0.64	0.30, 1.35	0.47	0.21, 1.07	0.89	0.24, 3.33
5 vs. 0	1.34	0.69, 2.59	1.13	0.54, 2.36	0.91	0.29, 2.88
6 vs. 0	0.92	0.46, 1.83	0.60	0.20, 1.87	1.54	0.30, 7.82
7 vs. 0	1.06	0.73, 1.52	0.79	0.48, 1.30	0.74	0.32, 1.67

Note. The definition of ‘moderate activity’ is an activity that requires moderated physical effort and evokes a somewhat increased heart rate or breathing, and ‘vigorous activity’ is an activity that requires hard physical effort and evokes a large increase in heart rate or breathing. Euro-Quality of Life 5 Dimension, OR = odds ratio, and CI = confidence interval. ^a^ *p* < 0.05, ^b^ *p* < 0.01, and ^c^ *p* < 0.001.

**Table 4:** The impact of physical activity on EQ-5D scores in adult patients with diabetes in Korea (multivariate regression model; control for covariates).

	OLS Regression Model		Tobit Regression Model	
	Coef.	95% CI	Coef.	95% CI
Walking				
1 vs. 0	0.05 ^c^	(0.02, 0.08)	0.07 ^b^	(0.01, 0.12)
2 vs. 0	0.06 ^c^	(0.02, 0.09)	0.07 ^a^	(0.01, 0.13)
3 vs. 0	0.06 ^c^	(0.03, 0.09)	0.08 ^b^	(0.03, 0.13)
4 vs. 0	0.05 ^a^	(0.00, 0.10)	0.09 ^a^	(0.01, 0.18)
5 vs. 0	0.05 ^b^	(0.01, 0.08)	0.04	(−0.02, 0.10)
6 vs. 0	0.07 ^c^	(0.04, 0.10)	0.11 ^b^	(0.03, 0.18)
7 vs. 0	0.04 ^c^	(0.02, 0.07)	0.05 ^b^	(0.01, 0.09)
Moderate				
1 vs. 0	0.03 ^b^	(0.01, 0.06)	0.06 ^a^	(0.01, 0.12)
2 vs. 0	0.02	(−0.01, 0.04)	0.01	(−0.05, 0.06)
3 vs. 0	0.02 ^a^	(0.00, 0.05)	0.05	(−0.01, 0.10)
4 vs. 0	0.02	(−0.01, 0.05)	0.04	(−0.04, 0.11)
5 vs. 0	0.00	(−0.03, 0.04)	−0.03	(−0.13, 0.06)
6 vs. 0	0.05 ^b^	(0.01, 0.08)	0.13 ^b^	(0.04, 0.22)
7 vs. 0	0.02	(−0.00 *, 0.05)	0.04	(−0.01, 0.09)
Vigorous				
1 vs. 0	−0.04	(−0.08, 0.01)	−0.07	(−0.15, 0.01)
2 vs. 0	0.00	(−0.02, 0.02)	−0.01	(−0.07, 0.05)
3 vs. 0	−0.03	(−0.05, 0.00)	−0.06	(−0.13, 0.00)
4 vs. 0	0.01	(−0.02, 0.05)	−0.00 *	(−0.12, 0.12)
5 vs. 0	−0.02	(−0.06, 0.02)	−0.06	(−0.15, 0.03)
6 vs. 0	0.02	(−0.03, 0.07)	0.02	(−0.09, 0.13)
7 vs. 0	−0.01	(−0.05, 0.03)	−0.00	(−0.08, 0.07)
Age (≥65 vs. <65)	−0.05 ^c^	(−0.07, −0.04)	−0.08 ^c^	(−0.11, −0.05)
Sex (female vs. male)	−0.01	(−0.03, 0.01)	−0.02	(−0.05, 0.01)
Region (city vs. non-city)	0.02 ^a^	(0.00, 0.03)	0.04 ^a^	(0.01, 0.07)
House income (reference: higher)				
Low	−0.07 ^c^	(−0.10, −0.05)	−0.13 ^c^	(−0.18, −0.08)
Lower middle	−0.01	(−0.03, 0.01)	−0.05 ^a^	(−0.09, −0.00 *)
Upper middle	−0.00 *	(−0.02, 0.01)	−0.02	(−0.06, 0.03)
Education (reference: college or higher)				
Elementary school or lower	−0.04 ^c^	(−0.06, −0.02)	−0.10 ^c^	(−0.15, −0.05)
Middle school	−0.01	(−0.03, 0.01)	−0.05	(−0.11, 0.00)
High school	−0.01	(−0.03, 0.01)	−0.03	(−0.08, 0.03)
Comorbidity				
Cardiovascular comorbidity	−0.02 ^a^	(−0.03, −0.00 *)	−0.04 ^a^	(−0.07, −0.01)
Musculoskeletal comorbidity	−0.08 ^c^	(−0.10, −0.06)	−0.13 ^c^	(−0.17, −0.10)
Respiratory comorbidity	−0.03 ^a^	(−0.05, −0.01)	−0.05 ^b^	(−0.09, −0.01)
Endocrine comorbidity	0.01	(−0.03, 0.04)	0.02	(−0.04, 0.08)
Cancer comorbidity	0.01	(−0.01, 0.04)	0.00	(−0.05, 0.05)
Mental comorbidity	−0.07 ^c^	(−0.10, −0.05)	−0.11 ^c^	(−0.15, −0.08)
Skin comorbidity	0.03	(−0.01, 0.06)	0.05	(−0.02, 0.13)
Urologic comorbidity	−0.11 ^a^	(−0.20, −0.02)	−0.16 ^b^	(−0.26, −0.05)
Digestive comorbidity	0.00	(−0.03, 0.03)	−0.03	(−0.09, 0.04)
Constant	0.94	(0.90, 0.96)	1.14	(1.07, 1.12)

Note. EQ-5D = Euro-Quality of Life 5 Dimension, OLS = ordinary least squares, Coef = coefficient, and CI = confidence interval. ^a^ *p* < 0.05, ^b^ *p* < 0.01, and ^c^ *p* < 0.001. * = significant difference of multivariate regression model of controlling for covariates.

## Data Availability

The datasets generated and examined for this study are not publicly accessible, but they can be obtained from the corresponding author upon reasonable request.
